# Dietary Habits and Osteoporotic Fracture Risk: Retrospective Cohort Study Using Large-Scale Claims Data

**DOI:** 10.1210/jendso/bvaf127

**Published:** 2025-08-28

**Authors:** Hiroki Nakajima, Yuichi Nishioka, Yuko Tamaki, Fumika Kamitani, Yukako Kurematsu, Sadanori Okada, Tomoya Myojin, Tatsuya Noda, Tomoaki Imamura, Yutaka Takahashi

**Affiliations:** Department of Diabetes and Endocrinology, Nara Medical University, Nara 634-8521, Japan; Department of Diabetes and Endocrinology, Nara Medical University, Nara 634-8521, Japan; Department of Public Health, Health Management and Policy, Nara Medical University, Nara 634-8521, Japan; Department of Diabetes and Endocrinology, Nara Medical University, Nara 634-8521, Japan; Department of Diabetes and Endocrinology, Nara Medical University, Nara 634-8521, Japan; Department of Diabetes and Endocrinology, Nara Medical University, Nara 634-8521, Japan; Department of Diabetes and Endocrinology, Nara Medical University, Nara 634-8521, Japan; Department of Public Health, Health Management and Policy, Nara Medical University, Nara 634-8521, Japan; Department of Public Health, Health Management and Policy, Nara Medical University, Nara 634-8521, Japan; Department of Public Health, Health Management and Policy, Nara Medical University, Nara 634-8521, Japan; Department of Diabetes and Endocrinology, Nara Medical University, Nara 634-8521, Japan

**Keywords:** osteoporotic fracture, lifestyle, dietary habit, skipping breakfast, late dinner

## Abstract

**Context:**

Lifestyle habits, such as exercise, alcohol consumption, and smoking, are known to be closely associated with the risk of osteoporotic fracture. However, little is known regarding the association between osteoporotic fracture and dietary habits such as skipping breakfast and having a late dinner.

**Objective:**

This study aimed to examine the association between lifestyle habits, including diet, and the risk of osteoporotic fracture.

**Methods:**

Individuals aged 20 years or older were enrolled using the results of lifestyle questionnaires in health checkup data and the DeSC database, a Japanese claims database. Outcome was defined as the diagnosis of osteoporotic fracture (hip, distal forearm, vertebral, and humeral fractures). A Cox proportional-hazards model was used to calculate the association between osteoporotic fracture risk and lifestyle, adjusting for conventional risk factors. In the lifestyle questionnaires, those who answered “yes” to each question were compared to those who answered “no.”

**Results:**

Altogether, 927 130 participants were included, with a median follow-up duration of 2.6 years. The adjusted hazard ratios (95% CI) for lifestyle factors of smoking, daily alcohol consumption, exercise habits, fast gait speed, enough sleep, skipping breakfast, and late dinner were 1.11 (1.06-1.17), 0.91 (0.88-0.95), 0.99 (0.97-1.02), 0.84 (0.82-0.86), 0.95 (0.93-0.98), 1.18 (1.12-1.23), and 1.08 (1.04-1.12), respectively.

**Conclusion:**

Our study is the first to demonstrate that skipping breakfast and having a late dinner are independently associated with a higher risk of osteoporotic fracture, using a large health checkup cohort.

Osteoporosis is a serious public health concern, and prevention of osteoporotic fractures is a crucial concern [1]. Risk factors for osteoporosis include age, weight, sex, family history, medications, and lifestyle [2], of which, lifestyle habits are modifiable and may enable the prevention of osteoporotic fractures. Lifestyle factors such as lack of exercise, lack of sleep, smoking, and heavy alcohol consumption have been identified as risks for osteoporosis [3-7], whereas there are few reports on its association with dietary habits such as skipping breakfast and having a late dinner.

Among dietary habits, skipping breakfast and having late dinner are associated with obesity, type 2 diabetes, and cardiovascular diseases [8-11]. Skipping breakfast is associated with lower bone mineral density (BMD), and those who reported skipping breakfast, in the questionnaire, had higher odds ratios of fracture, without osteoporosis [12, 13]. However, a randomized mendelian analysis found no association between skipping breakfast and BMD [14]. Few reports have examined the association between skipping breakfast and osteoporotic fractures longitudinally, and no studies have examined the association between osteoporosis and late dinner.

Regarding lifestyle factors beyond dietary habits, smoking has been associated with decreased BMD and an increased risk of hip and lumbar spine fractures [15]. For alcohol consumption, previous studies have reported that fracture risk varies by fracture site and alcohol intake level [16]. Physical activity has been shown to enhance BMD in the lumbar spine and femoral neck [7]. Sleep duration has also been reported to be associated with BMD and osteoporosis risk [17]. However, few studies have conducted stratified analyses of osteoporotic fractures, and data on distal forearm and proximal humerus fractures remain limited because of the small sample sizes in previous studies.

In this study, we investigated the association of skipping breakfast and having a late dinner with osteoporotic fractures, using a large-scale claims database containing health checkup data of approximately 11 million people in Japan [18]. Additionally, we investigated the relationship between lifestyle factors other than dietary habits and the risk of osteoporotic fractures.

## Materials and Methods

### Study Design

**Figure 1.**
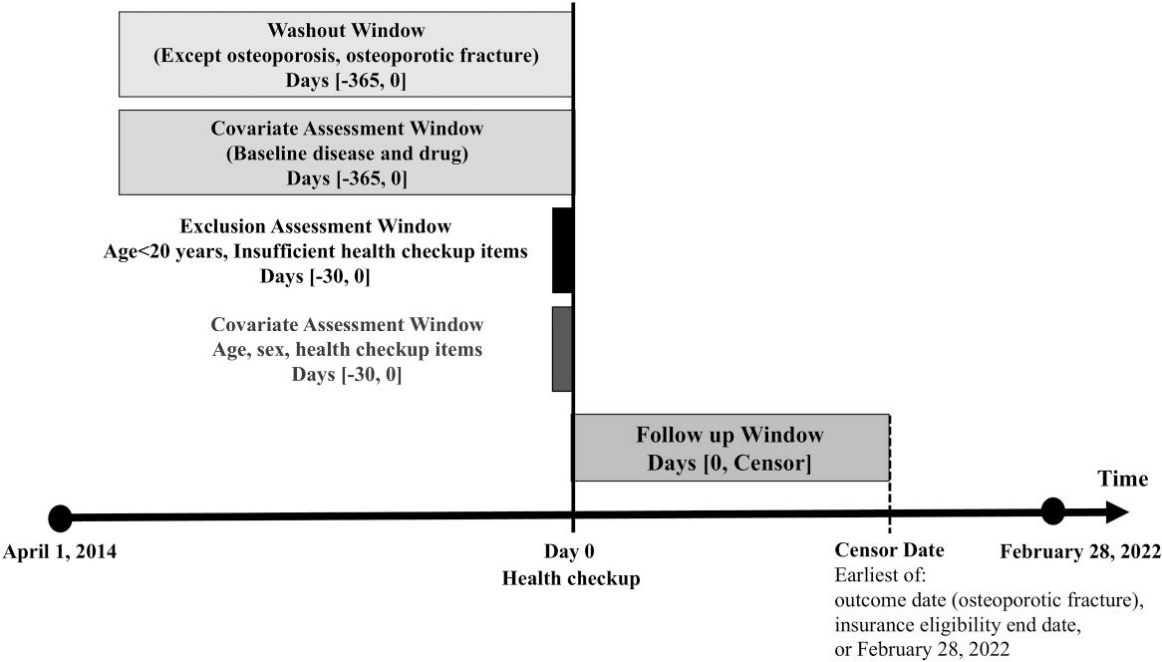
Study design.

This cohort study was approved by the Nara Medical University Ethics Committee (approval No. 1123-8) and anonymized data from the DeSC database, which contains information on claims and health checkups in Japan, was used. Since all data in this database are anonymized, the requirement for informed consent has been waived. We enrolled individuals aged 20 years or older who were registered in the database and underwent a health checkup between April 1, 2014, and February 28, 2022. The observation start date was the time of the health checkup visit. The washout period was defined as the period from 365 days prior to the health checkup date, to extract the comorbidities and medications used as of the start date of the observation (Fig. 1). We enrolled individuals with information on their physical data and lifestyle questionnaire items during the health checkup. The questionnaire was administered at the observation start date. Patients diagnosed with osteoporosis or osteoporotic fractures during the washout period were excluded. The censoring date was set as the earliest date among the outcome date, insurance eligibility end date, or February 28, 2022 (see Fig. 1).

### Data Sources

The DeSC database is Japan's claims database and consists of claims and health checkup data collected from several health insurance societies, including the Health Insurance Society, National Health Insurance Society, and Wide-area Federation of Health Care for Later-Stage Senior Citizens [18].

Claims data in the DeSC database contain records of disease name diagnoses, medical procedures, and drugs used by all patients during the insurance period. Approximately 20% of registrants in the DeSC database had health checkup data. Medical examinations included sex, date of birth, height, weight, blood pressure, and a lifestyle questionnaire (Supplementary Table S1) [19]. In Japan, the most common dietary habit is having 3 meals a day: breakfast, lunch, and dinner. The questionnaire used in this study was based on the Specific Health Checkups and Specific Health Guidance, a nationwide health screening and guidance program introduced by the Japanese government in 2008, and has been used in various studies [19-22].

We defined skipping breakfast (+) as those who answered “Yes” to the question “Do you skip breakfast more than 3 times a week?” in the questionnaire, and skipping breakfast (−) as those who answered “No.” We defined late dinner (+) as those who answered “Yes” to the question “Do you eat supper less than 2 hours before bedtime more than 3 times a week?” in the questionnaire, and late dinner (−) as those who answered “No.” Questions regarding other habits (weight gain after age 20, exercise habit, physical activity, fast gait speed, and enough sleep) were answered with “Yes” or “No” as well. The questions on drinking habits had “every day” or “sometimes” or “rarely drink (cannot drink)” as responses.

### Exposure

Lifestyle habits, including dietary habits, were assessed at the time of the health checkup. Exposure was defined as “Yes” to questions regarding lifestyle habits except drinking habits. In other words, exposure was defined as a “Yes” response to the questionnaire's “skipping breakfast,” “late dinner,” “weight gain after age 20,” “exercise habit,” “physical activity,” “fast gait speed,” or “enough sleep.” Drinking habits were considered as exposure if the participants answered “every day” and “sometimes.”

### Outcomes

The primary outcome was the date of occurrence of major osteoporotic fracture (hip, distal forearm, vertebra, or humerus), as recorded in the insurance claims. Major osteoporotic fractures are described as a fracture risk index of clinical risk factors (FRAX), such as a fracture of the hip, distal antebrachium, vertebrae, or humerus [23]. In a similar study using claims database, the primary endpoint of osteoporotic fractures was defined using the International Classification of Diseases 10th Revision (ICD-10) codes based on FRAX and defined as fractures of the hip, distal antebrachium, vertebrae, or humerus [24]. Therefore, we used the same definition for the disease codes corresponding to the ICD-10 as previously described [24]. The correspondence between each disease name and ICD-10 is shown in Supplementary Table S2 [2, 24]. The secondary outcomes were defined as the occurrence of fractures exclusively at the hip, distal forearm, vertebrae, or humerus.

### Statistical Analysis

Statistical analyses were performed using the IBM SPSS Statistics 29.0.1. Normally distributed continuous variables are presented as mean and SD, while continuous variables that were not normally distributed are presented as medians and quartiles. Categorical variables were calculated as percentages within groups. Categorical variables were analyzed using the chi-square test or Bonferroni correction, while continuous variables were compared using the Kruskal-Wallis test followed by Bonferroni correction. We calculated the incidence rate per 1000 person-years of the outcomes. We estimated the adjusted hazard ratios (aHR) for “skipping breakfast” and “late dinner” using the Cox proportional-hazards model with adjustment for covariates for primary or secondary outcomes. The covariates included other lifestyle habits such as “weight gain after age 20,” “exercise habit,” “physical activity,” “fast gait speed,” “enough sleep,” and “drinking habits”, age, sex, body mass index (BMI), systolic blood pressure, diastolic blood pressure, blood tests, proteinuria, disease name, and drug use (Supplementary Table S3) [2, 19]. A Cox proportional-hazards model adjusted for covariates was used to analyze the combination of skipping breakfast and having a late dinner. “Skipping breakfast (−) + late dinner (−)” was used as reference for this analysis. The covariates included the same variables as previously described. The covariates for disease and medications were those related to hypertension, diabetes, dyslipidemia, and secondary osteoporosis. Items related to secondary osteoporosis were selected based on the American Association of Clinical Endocrinologists and American College of Endocrinology Clinical Practice Guidelines [2]. Disease codes based on the ICD-10 were used for diseases, and drug codes applied in Japan were used for drugs. Regarding steroid administration, the covariates were prednisolone 5 mg or more for at least 3 months and betamethasone/d-chlorpheniramine maleate 2 or more tablets for at least 3 months, according to the FRAX score.

## Results

**Figure 2.**
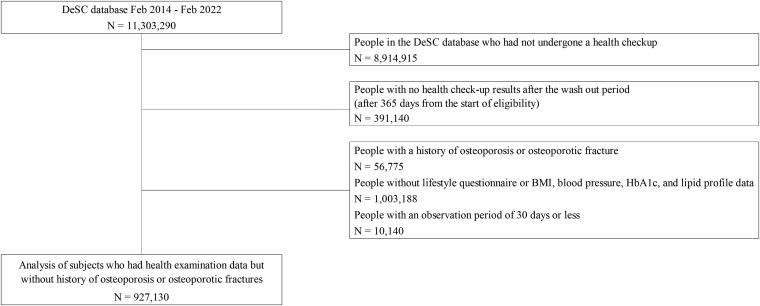
Flowchart of the study.

We identified 927 130 individuals who met the inclusion criteria (Fig. 2). The median age at the start of observation was 66.6 years (interquartile range, 57.6-70.3 years). The median follow-up was 2.6 years (interquartile range, 1.4-4.3 years), and the number of major osteoporotic fractures was 28 196 with an incidence rate of 10.8 per 1000 person-years during the observation period. The number of hip, distal forearm, vertebral, and humeral fractures were 3382, 6983, 15 922, and 4213, respectively. The incidence rates per 1000 person-years for hip, distal forearm, vertebral, and humeral fractures were 1.3, 2.6, 6.1, and 1.6, respectively. Table 1 shows the general characteristics of the participants and the 4 groups divided by dietary habits (1. “skipping breakfast (−) + late dinner (−),” 2. “skipping breakfast (−) + late dinner (+),” 3. “skipping breakfast (+) + late dinner (−),” 4. “skipping breakfast (+) + late dinner (+)”) at baseline. Using the Bonferroni correction, statistically significant differences were found among all dietary habit groups regarding sex, smoking, drinking habits, weight gain after age 20, exercise habits, physical activity, fast gait speed, and sufficient sleep. Age also differed significantly across groups. For BMI, statistically significant differences were observed among all groups, except between the “skipping breakfast (−) + late dinner (+)” and “skipping breakfast (+) + late dinner (+)” groups. The “skipping breakfast (−) + late dinner (−)” group was the oldest, with a median age of 67.2 years, and the “skipping breakfast (+) + late dinner (+)” group was the youngest, with a median age of 52.3 years. In terms of BMI, the “skipping breakfast (−) + late dinner (−)” group had the lowest value with a median BMI of 22.9 and the “skipping breakfast (+) + late dinner (+)” group had the highest with a median BMI of 23.7. The “skipping breakfast (+) + late dinner (+)” group had the highest proportion of current smokers (42.6%) and daily drinkers (56.3%), and the lowest proportion of individuals with regular exercise habits (23.2%) and sufficient sleep (56.3%) compared with the other groups.

**Table 1.** Baseline characteristics of participants grouped by dietary habits

	Total	Skipping breakfast (−), late dinner (−) (ref)	Skipping breakfast (−), late dinner (+)		Skipping breakfast (+), late dinner (−)		Skipping breakfast (+), late dinner (+)	
	n = 927 130	n = 710 824	n = 125 123		n = 55 603		n = 35 580	
Age, median (IQR), y	66.6 (57.6-70.3)	67.2 (61.3-70.6)	64.9 (50.9-70.1)	<0.001	57.8 (45.7-67.5)	<0.001	52.3 (42.7-63.3)	<0.001
Sex, %								
Male	45.3	40.8	60.5	<0.001	54.0	<0.001	68.5	<0.001
Female	54.7	59.2	39.5	<0.001	46.0	<0.001	31.5	<0.001
BMI, mean (SD)	23.1 ± 3.6	22.9 ± 3.5	23.6 ± 3.7	<0.001	23.3 ± 14.0	<0.001	23.7 ± 4.0	<0.001
Daily habit								
Current smoker, %	14.4	10.5	20.2	<0.001	33.4	<0.001	42.6	<0.001
Drinking habits, %								
Rarely or does not drink	54.8	58.6	40.5	<0.001	49.6	<0.001	37.8	<0.001
Sometimes	22.2	21.7	22.6	<0.001	25.1	<0.001	26.1	<0.001
Everyday	23.0	19.7	36.9	<0.001	25.3	<0.001	37.8	<0.001
Weight gain after age 20, %	33.9	31.7	20.2	<0.001	39.0	<0.001	45.6	<0.001
Exercise habits, %	38.0	40.1	35.9	<0.001	25.2	<0.001	23.2	<0.001
Physical activity, %	49.7	50.7	49.5	<0.001	41.4	<0.001	42.1	<0.001
Fast gait speed, %	46.8	47.4	45.9	<0.001	42.4	<0.001	44.1	<0.001
Enough sleep, %	72.4	74.5	67.4	<0.001	66.7	<0.001	56.3	<0.001
Systolic BP, mm Hg, mean (SD)	129.2 ± 18.4	129.6 ± 18.4	129.1 ± 18.4	<0.001	126.6 ± 18.9	<0.001	126.1 ± 18.5	<0.001
Diastolic BP, mm Hg, mean (SD)	75.6 ± 11.5	75.5 ± 11.3	76.1 ± 11.7	<0.001	76.0 ± 12.2	<0.001	76.7 ± 12.3	<0.001

Abbreviations: BMI, body mass index; BP, blood pressure; IQR, interquartile range; ref, reference.

### Osteoporotic Fractures Associated With Lifestyle

**Figure 3.**
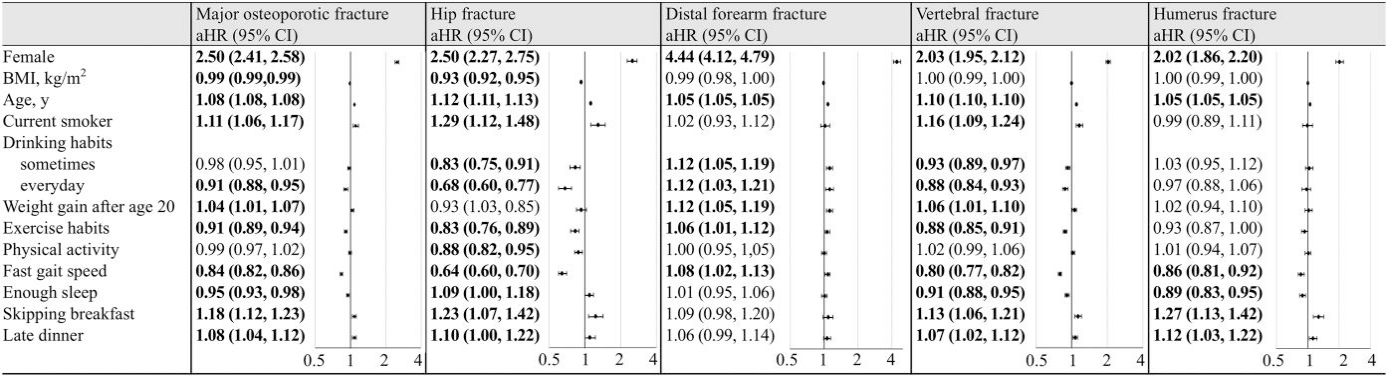
Hazard ratios for osteoporotic fractures based on sex, age, BMI, and different lifestyle habits. The aHR were calculated by adjusting for all covariates (age, sex, BMI, lifestyle questionnaire, systolic blood pressure, diastolic blood pressure, blood test, proteinuria, disease name, and medication). Bold text indicates statistical significance. aHR, adjusted hazard ratios; BMI, body mass index.

Fig. 3 shows the association between risk of osteoporotic fractures and lifestyle after adjusting for conventional risk factors. In terms of dietary habits, even after adjusting for conventional risk factors, skipping breakfast resulted in a significantly higher aHR of 1.18 (95% CI, 1.12-1.23). Late dinner also showed a significantly higher aHR of 1.08 (95% CI, 1.04-1.12) for osteoporotic fractures. Among the osteoporotic fractures, skipping breakfast showed a significantly higher aHR for hip, vertebral, and humerus fractures (1.23 [95% CI, 1.07-1.42], 1.13 [95% CI, 1.06-1.21], and 1.27 [95% CI, 1.13-1.42], respectively). Similarly, late dinner had a significantly higher aHR for hip fracture, vertebral fracture, and humerus fracture (1.10 [95% CI, 1.00-1.22], 1.07 [95% CI, 1.02-1.12], and 1.12 [95% CI, 1.03-1.22], respectively). Distal forearm fractures were not associated with skipping breakfast or having a late dinner.

Smoking resulted in a significantly higher aHR of 1.11 (95% CI, 1.06-1.17) for osteoporotic fracture. Drinking every day, exercise habits, and fast walking speed had significantly lower aHR for osteoporotic fracture (0.91 [95% CI, 0.88-0.95], 0.91 [95% CI, 0.89-0.94], and 0.84 [95% CI, 0.82-0.86], respectively), significantly lower aHR for hip fracture and vertebral fracture, but were associated with an increased aHR of 1.12 (95% CI, 1.05-1.19), 1.06 (95% CI, 1.01-1.12), and 1.08 (95% CI, 1.02-1.13), respectively, for distal forearm fracture. Daily drinking habit was associated with a significantly lower aHR for osteoporotic fractures, whereas the disease name of “mental and behavioral disorders due to alcohol use” exhibited a significantly higher aHR of 1.80 (95% CI, 1.44-2.25) for osteoporotic fractures. Weight gain after age 20 years showed a significantly higher aHR of 1.04 (95% CI, 1.01-1.07) for osteoporotic fractures. Enough sleep was associated with decreased aHR of 0.95 in osteoporotic fracture (95% CI, 0.93-0.98).

### General Risk Factors for Osteoporotic Fractures

Fig. 3 shows the aHR based on age, sex, BMI, and habits after adjusting for conventional risk factors. Women showed significantly higher aHRs of 2.50 (95% CI, 2.41-2.58) for osteoporotic fractures. Among fractures, those of the distal forearm had the highest aHR of 4.44 (95% CI, 4.12-4.79). An increased BMI was associated with a significantly lower aHR of 0.99 for osteoporotic fracture (95% CI, 0.99-0.99). Increased age was associated with a significantly higher aHR of 1.08 (95% CI, 1.08-1.08) for osteoporotic fracture.

### Osteoporotic Fractures Associated With Combinations of Dietary Habits

**Figure 4.**
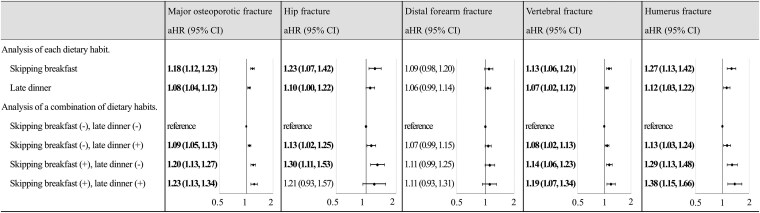
Hazard ratios for osteoporotic fracture based on breakfast and dinner habits and its combination. Regression analysis was performed independently for “Analysis of each dietary habit” and “Analysis of a combination of dietary habits. “Skipping breakfast (−) + late dinner (−)” was used as reference for the analysis of combination of dietary habits. The aHR for “each dietary habit” was calculated using a model adjusted for all covariates (age, sex, BMI, lifestyle questionnaire, systolic blood pressure, diastolic blood pressure, blood tests, proteinuria, disease name, and drugs). The aHR for the “combination of dietary habits” item are calculated from a model adjusting for all covariates (age, sex, BMI, lifestyle questionnaire, systolic blood pressure, diastolic blood pressure, blood test, proteinuria, disease name, and medications). The bold text indicates statistical significance. aHR, adjusted hazard ratios; BMI, body mass index.

Next, we analyzed the effect of a combination of dietary habits on osteoporotic fractures (Fig. 4). The aHR for each combination of “skipping breakfast (−) + late dinner (+),” “skipping breakfast (+) + late dinner (−),” and “skipping breakfast (+) + late dinner (+)” compared to “skipping breakfast (−) + late dinner (−),” were 1.09 (95% CI, 1.05-1.13), 1.20 (95% CI, 1.13-1.27), and 1.23 (95% CI, 1.13-1.34), respectively. These data clearly demonstrate that the combination of “skipping breakfast (+) + late dinner (+)” resulted in an additively higher aHR of osteoporotic fractures.

## Discussion

In this study, we used large-scale claims database with lifestyle questionnaires on health checkup data and clarified the effect of dietary habits on osteoporotic fracture risk. In addition, we demonstrated the effect not only on overall osteoporotic fractures but also on each fracture site in detail. We newly demonstrated longitudinally that skipping breakfast and having a late dinner were independently associated with an increased risk of osteoporotic fractures, along with conventional risks. We showed a higher risk of osteoporotic fractures in women with lower BMI and older age, which is consistent with previous reports and demonstrates the validity of this cohort study. We also showed that dietary habits additively increased the risk. Notably, the dietary habits of skipping breakfast and eating a late dinner were associated with young age, smoking, alcohol consumption, less exercise, and less sleep, suggesting that these unhealthy lifestyle habits can accumulate. These data clearly indicate that osteoporotic fracture is lifestyle related, which includes dietary habits.

Our study is the first to demonstrate that late dinner habits are associated with a higher risk of osteoporotic fracture. Late dinners, compared to early dinners, have reportedly been associated with increased blood glucose levels, decreased nocturnal fatty acid concentrations, elevated cortisol levels, and obesity [25-28]. Although these findings are based on studies related to time-restricted feeding, research has shown that a 12-hour eating window results in higher oxidative stress and lower insulin sensitivity compared to a 6-hour eating window, where dinner is consumed by 3 Pm [29]. Late dinners may contribute to elevated cortisol levels and oxidative stress. Increased glucocorticoid levels negatively affect bone health [30]. Endogenous glucocorticoids have been reported to affect skeletal fragility [31]. In humans, elevated oxidative stress is associated with decreased BMD [32]. Therefore, late dinners may lead to increased cortisol levels and oxidative stress, potentially affecting bone metabolism and contributing to the risk of osteoporosis [30, 32].

There are several reports on skipping breakfast. A prospective cohort study of young Japanese individuals showed that men who skipped breakfast exhibited a decrease in lumbar spine BMD after 3 years [13]. In another cross-sectional study on Japanese female university students, skipping breakfast was associated with lower hip bone density [33]. In postmenopausal women, the odds ratio of fracture in the breakfast-skipping group was 2.3-fold more after 2 years [12]; there was no information on medications or medical history, and the outcomes of osteoporosis and fracture were reported by the patients. In our study, we used the DeSC claims database, which contains the names of diseases and medications. In addition, the date of fracture was available, which enabled the evaluation of longitudinal risk. A recent study reported that no relationship was detected between skipping breakfast and BMD of the forearm, femoral neck, and lumbar spine, using mendelian randomization [14]; however, the association with osteoporotic fracture was not clarified. Our study clearly demonstrated that skipping breakfast and having a late dinner were independently and additively associated with an increased risk of fractures.

Regarding the mechanism underlying these dietary habits, those who skipped breakfast had lower vitamin D and calcium intake [34] and lower serum 25-hydroxyvitamin D levels than in those who ate breakfast [35], suggesting that vitamin D deficiency due to a lack of breakfast intake may influence the increased risk of osteoporotic fractures. There are very few studies that have examined the extent to which skipping breakfast affects vitamin D intake among the Japanese population; however, consuming fish or mushrooms, which are rich in vitamin D, is not common for breakfast [36]. Therefore, the effect of skipping breakfast on vitamin D levels may be limited. Osteoblasts, osteoclasts, and osteocytes mostly express circadian clock genes, and it has been reported that disruption of circadian rhythms induced a decrease in bone mass and density [37]. Diet is a powerful stimulator that affects the phase of the peripheral clock [38], and breakfast affects the clock and clock-regulated genes [39]. Disordered rhythms associated with these dietary habits may influence BMD. Indeed, individuals who skipped breakfast and had a late dinner showed a higher ratio of “not enough sleep” in this study, suggesting an association with disordered rhythm.

With regard to alcohol consumption, a meta-analysis showed an increase in BMD at the lumbar spine or femoral neck with standard alcohol consumption compared to no drinking habits and that heavy drinking increases the risk of osteoporotic fractures [5]. In our study, the risk of osteoporotic fractures was higher if the patients had the disease name “mental and behavioral disorders due to use of alcohol,” which is consistent with previous reports. Interestingly, daily alcohol consumption was associated with a higher risk of distal forearm fracture but a lower risk of other osteoporotic fracture sites and an overall lower risk of osteoporotic fracture, suggesting that daily alcohol consumption can affect the risk depending of the site of osteoporotic fracture and the background.

We showed that those who reported “exercise habit,” “fast walking speed,” and “enough sleep” had lower risk of osteoporotic fractures. Earlier studies have shown that exercise habit is associated with a lower risk of osteoporosis [7], and self-reported fast walking speed is associated with increased bone mass [3], which is compatible with our results. Regarding the site of osteoporotic fractures, those who reported “everyday drinking habit,” “exercise habit,” and “fast walking speed,” had a lower risk of overall osteoporotic fracture; however, distal forearm fracture showed an increased risk. These data suggest that distal forearm fractures involve different pathological conditions. It has been reported that the peak age of occurrence of distal radius fractures is 5 to 10 years earlier than that of vertebral and femoral neck fractures [40], suggesting that distal forearm fractures may be associated with a younger age and relatively active activities of daily living conditions.

This study had several limitations. First, because the washout period was 1 year, history of osteoporotic fractures prior to the washout period was not evaluated. However, because individuals taking medications for osteoporosis were excluded, patients with osteoporosis who received medical treatment were also excluded. Second, family history of osteoporosis was not adjusted for because it was not available in the DeSC database. Third, the lifestyle questionnaire was self-administered and primarily encompassed binary (yes/no) response options, except for alcohol consumption. Given the subjective nature of these assessments and the lack of quantitative measures, future research should incorporate both objective and quantitative methodologies. Fourth, the outcome of osteoporotic fractures in this study could not distinguish fractures caused by high-energy trauma, such as falls from a height or traffic accidents. Therefore, further studies are needed to investigate the relationship between these dietary habits and the detailed causes of fractures. Nevertheless, this study has several strengths. Previous studies on osteoporosis and dietary habits have included a maximum of 70 000 individuals; however, this study included a total of 900 000 participants, using this large-scale claims database. This allowed us to perform a detailed site-specific analysis of osteoporotic fractures and adjust for an adequate number of covariates.

In conclusion, our study is the first to suggest that skipping breakfast and having a late dinner were independently associated with a higher risk of osteoporotic fracture, in addition to the conventional risk factors, using a large-scale claims database. These results indicated that osteoporosis is a lifestyle-related disease. Future research is needed to investigate the relationship between late-night dinners and bone metabolism, as well as intervention studies focusing on guidance regarding skipping breakfast and having late dinners.

## Data Availability

All data sets analyzed during the current study are not publicly available but are available from the corresponding author on reasonable request.

## Abbreviations

aHR: adjusted hazard ratio
BMD: bone mineral density
BMI: body mass index
FRAX: fracture risk index of clinical risk factors
ICD-10: International Classification of Diseases 10th Revision

## References

[1] NIH Consensus Development Panel on Osteoporosis Prevention D, and Therapy . Osteoporosis prevention, diagnosis, and therapy. JAMA. 2001;285(6):785‐795. 11176917 10.1001/jama.285.6.785

[2] Watts NB, Camacho PM, Lewiecki EM, Petak SM. American Association of Clinical Endocrinologists/American College of Endocrinology Clinical Practice Guidelines for the diagnosis and treatment of postmenopausal osteoporosis-2020 update. Endocr Pract. 2021;27(4):379‐380. 33577971 10.1016/j.eprac.2021.02.001

[3] Tomita Y, Arima K, Mizukami S, et al Association between self-reported walking speed and calcaneal stiffness index in postmenopausal Japanese women. BMC Geriatr. 2020;20(1):466. 33176711 10.1186/s12877-020-01858-4 PMC7661156

[4] Tang Y, Wang S, Yi Q, Xia Y, Geng B. Sleep pattern and bone mineral density: a cross-sectional study of National Health and Nutrition Examination Survey (NHANES) 2017–2018. Arch Osteoporos. 2021;16(1):157. 34689259 10.1007/s11657-021-01025-1

[5] Godos J, Giampieri F, Chisari E, et al Alcohol consumption, bone mineral density, and risk of osteoporotic fractures: a dose–response meta-analysis. Int J Environ Res Public Health. 2022;19(3):1515. 35162537 10.3390/ijerph19031515 PMC8835521

[6] LeBoff MS, Greenspan SL, Insogna KL, et al The clinician's guide to prevention and treatment of osteoporosis. Osteoporos Int. 2022;33(10):2049‐2102. 35478046 10.1007/s00198-021-05900-y PMC9546973

[7] Zhang S, Huang X, Zhao X, et al Effect of exercise on bone mineral density among patients with osteoporosis and osteopenia: a systematic review and network meta-analysis. J Clin Nurs. 2022;31(15-16):2100‐2111. 34725872 10.1111/jocn.16101

[8] Cahill LE, Chiuve SE, Mekary RA, et al Prospective study of breakfast eating and incident coronary heart disease in a cohort of male US health professionals. Circulation. 2013;128(4):337‐343. 23877060 10.1161/CIRCULATIONAHA.113.001474 PMC3797523

[9] Davis R, Rogers M, Coates AM, Leung GKW, Bonham MP. The impact of meal timing on risk of weight gain and development of obesity: a review of the current evidence and opportunities for dietary intervention. Curr Diab Rep. 2022;22(4):147‐155. 35403984 10.1007/s11892-022-01457-0 PMC9010393

[10] Mekary RA, Giovannucci E, Cahill L, Willett WC, van Dam RM, Hu FB. Eating patterns and type 2 diabetes risk in older women: breakfast consumption and eating frequency. Am J Clin Nutr. 2013;98(2):436‐443. 23761483 10.3945/ajcn.112.057521 PMC3712552

[11] Wicherski J, Schlesinger S, Fischer F. Association between breakfast skipping and body weight-A systematic review and meta-analysis of observational longitudinal studies. Nutrients. 2021;13(1):272. 33477881 10.3390/nu13010272 PMC7832891

[12] Otonari J, Ikezaki H, Furusyo N, Sudo N. Association of lifestyle factors with osteoporosis and fracture in postmenopausal women: a Japanese cohort study. Menopause. 2021;28(11):1254‐1263. 34313618 10.1097/GME.0000000000001840

[13] Nagata K, Yoshida M, Ishimoto Y, Hashizume H, Yamada H, Yoshimura N. Skipping breakfast and less exercise are risk factors for bone loss in young Japanese adults: a 3-year follow-up study. J Bone Miner Metab. 2014;32(4):420‐427. 24052206 10.1007/s00774-013-0510-5

[14] Yu J, Zhuang C, Guo W, et al Causal relationship between breakfast skipping and bone mineral density: a two-sample Mendelian randomized study. Front Endocrinol (Lausanne). 2023;14:1200892. 38027166 10.3389/fendo.2023.1200892 PMC10660815

[15] Weng W, Li H, Zhu S. An overlooked bone metabolic disorder: cigarette smoking-induced osteoporosis. Genes (Basel). 2022;13(5):806. 35627191 10.3390/genes13050806 PMC9141076

[16] Wang SM, Han KD, Kim NY, et al Association of alcohol intake and fracture risk in elderly varied by affected bones: a nationwide longitudinal study. Psychiatry Investig. 2020;17(10):1013‐1020. 10.30773/pi.2020.0143 PMC7596281 33059395

[17] Tian J, Zhang J, Ding L, Qi X. Association between sleep duration and low bone mineral density and osteoporosis: a systematic review and meta-analysis. Calcif Tissue Int. 2024;116(1):3. 39673612 10.1007/s00223-024-01319-9

[18] Okada A, Yasunaga H. Prevalence of noncommunicable diseases in Japan using a newly developed administrative claims database covering young, middle-aged, and elderly people. JMA J. 2022;5(2):190‐198. 35611228 10.31662/jmaj.2021-0189 PMC9090547

[19] Tsushita K, SH A, Miura K, et al Rationale and descriptive analysis of specific health guidance: the nationwide lifestyle intervention program targeting metabolic syndrome in Japan. J Atheroscler Thromb. 2018;25(4):308‐322. 29238010 10.5551/jat.42010 PMC5906184

[20] Hidaka T, Kasuga H, Endo S, et al Are lifestyle pattern changes associated to poor subjective sleep quality? : a cross-sectional study by gender among the general Japanese population underwent specified medical check-ups in 2014 and 2015. BMJ Open. 2020;10(12):e037613. 10.1136/bmjopen-2020-037613 PMC7745693 33328256

[21] Iwasaki M, Kudo A, Asahi K, et al Fast walking is a preventive factor against new-onset diabetes mellitus in a large cohort from a Japanese general population. Sci Rep. 2021;11(1):716. 33436978 10.1038/s41598-020-80572-y PMC7804125

[22] Kitazawa A, Maeda S, Fukuda Y. Fatty liver index as a predictive marker for the development of diabetes: a retrospective cohort study using Japanese health check-up data. PLoS One. 2021;16(9):e0257352. 34543321 10.1371/journal.pone.0257352 PMC8451989

[23] Kanis JA, Johnell O, Oden A, Johansson H, McCloskey E. FRAX and the assessment of fracture probability in men and women from the UK. Osteoporos Int. 2008;19(4):385‐397. 18292978 10.1007/s00198-007-0543-5 PMC2267485

[24] Egeberg A, Schwarz P, Harsløf T, et al Association of potent and very potent topical corticosteroids and the risk of osteoporosis and Major osteoporotic fractures. JAMA Dermatol. 2021;157(3):275‐282. 33471030 10.1001/jamadermatol.2020.4968 PMC7970335

[25] Nakajima K, Suwa K. Association of hyperglycemia in a general Japanese population with late-night-dinner eating alone, but not breakfast skipping alone. J Diabetes Metab Disord. 2015;14(1):16. 25874189 10.1186/s40200-015-0147-0 PMC4396539

[26] Gu C, Brereton N, Schweitzer A, et al Metabolic effects of late dinner in healthy volunteers-A randomized crossover clinical trial. J Clin Endocrinol Metab. 2020;105(8):2789‐2802. 32525525 10.1210/clinem/dgaa354 PMC7337187

[27] Nakamura K, Tajiri E, Hatamoto Y, Ando T, Shimoda S, Yoshimura E. Eating dinner early improves 24-h blood glucose levels and boosts lipid metabolism after breakfast the next day: a randomized cross-over trial. Nutrients. 2021;13(7):2424. 34371933 10.3390/nu13072424 PMC8308587

[28] Chauhan YV, Gada JV, Misra S, et al Early dinner improves the glycemic profile in habitual late eaters with uncontrolled type 2 diabetes Mellitus in the short term. Cureus. 2024;16(5):e59504. 38826926 10.7759/cureus.59504 PMC11144034

[29] Sutton EF, Beyl R, Early KS, Cefalu WT, Ravussin E, Peterson CM. Early time-restricted feeding improves insulin sensitivity, blood pressure, and oxidative stress even without weight loss in men with prediabetes. Cell Metab. 2018;27(6):1212‐1221.e3. 29754952 10.1016/j.cmet.2018.04.010 PMC5990470

[30] Seibel MJ, Cooper MS, Zhou H. Glucocorticoid-induced osteoporosis: mechanisms, management, and future perspectives. Lancet Diabetes Endocrinol. 2013;1(1):59‐70. 24622268 10.1016/S2213-8587(13)70045-7

[31] Weinstein RS, Wan C, Liu Q, et al Endogenous glucocorticoids decrease skeletal angiogenesis, vascularity, hydration, and strength in aged mice. Aging Cell. 2010;9(2):147‐161. 20047574 10.1111/j.1474-9726.2009.00545.x PMC2858771

[32] Basu S, Michaëlsson K, Olofsson H, Johansson S, Melhus H. Association between oxidative stress and bone mineral density. Biochem Biophys Res Commun. 2001;288(1):275‐279. 11594785 10.1006/bbrc.2001.5747

[33] Kuroda T, Onoe Y, Yoshikata R, Ohta H. Relationship between skipping breakfast and bone mineral density in young Japanese women. Asia Pac J Clin Nutr. 2013;22(4):583‐589. 24231019 10.6133/apjcn.2013.22.4.10

[34] O'Neil CE, Nicklas TA, Fulgoni VL. Nutrient intake, diet quality, and weight/adiposity parameters in breakfast patterns compared with no breakfast in adults: National Health and Nutrition Examination Survey 2001–2008. J Acad Nutr Diet. 2014;114(12):S27‐S43. 25458992 10.1016/j.jand.2014.08.021

[35] Fagnant HS, Lutz LJ, Nakayama AT, Gaffney-Stomberg E, McClung JP, Karl JP. Breakfast skipping is associated with vitamin D deficiency among young adults entering initial military training. J Acad Nutr Diet. 2022;122(6):1114‐1128.e1. 34601165 10.1016/j.jand.2021.09.016

[36] Ministry of Health, Labour and Welfare of Japan . The National Health and Nutrition Survey in Japan, 2019. Report in Japanese. Ministry of Health, Labour and Welfare of Japan. Accessed January 14, 2025. https://www.e-stat.go.jp/dbview?sid=0003234486

[37] Juliana N, Azmi L, Effendy NM, et al Effect of circadian rhythm disturbance on the human musculoskeletal system and the importance of nutritional strategies. Nutrients. 2023;15(3):734. 36771440 10.3390/nu15030734 PMC9920183

[38] Pickel L, Sung H-K. Feeding rhythms and the circadian regulation of metabolism. Front Nutr. 2020;7:39. 32363197 10.3389/fnut.2020.00039 PMC7182033

[39] Jakubowicz D, Wainstein J, Landau Z, et al Influences of breakfast on clock gene expression and postprandial glycemia in healthy individuals and individuals with diabetes: a randomized clinical trial. Diabetes Care. 2017;40(11):1573‐1579. 28830875 10.2337/dc16-2753

[40] Cuddihy MT, Gabriel SE, Crowson CS, O'Fallon WM, Melton LJ. Forearm fractures as predictors of subsequent osteoporotic fractures. Osteoporos Int. 1999;9(6):469‐475. 10624452 10.1007/s001980050172

